# Comparison of emergence agitation between tracheal tube and laryngeal mask airway in pediatric ophthalmic surgery: a propensity-score matching analysis

**DOI:** 10.3389/fmed.2026.1838626

**Published:** 2026-07-15

**Authors:** Jung A Lim, Jonghae Kim

**Affiliations:** 1 Department of Anesthesiology and Pain Medicine, School of Medicine, Kyungpook National University, Daegu, Republic of Korea; 2 Department of Anesthesiology and Pain Medicine, Daegu Catholic University Medical Center, Daegu Catholic University School of Medicine, Daegu, Republic of Korea

**Keywords:** airway management, emergence delirium, epiblepharon of upper lid, intratracheal intubation, laryngeal masks, strabismus

## Abstract

**Background:**

Although emergence agitation (EA) is common and can threaten patient safety in children undergoing ophthalmic surgery, large-scale studies examining the effects of airway management techniques on its development remain limited. This study aimed to compare the development of EA between tracheal tube and laryngeal mask airway (LMA) in pediatric ophthalmic surgery.

**Methods:**

We retrospectively analyzed the medical records of 1029 children aged 3–7 years who underwent strabismus or epiblepharon surgery between January 2014 and December 2022 in Daegu Catholic University Medical Center, Daegu, Republic of Korea. Patients were assigned to either the tracheal tube or LMA groups and matched in a 1:1 ratio using propensity scores. Multivariable logistic regression analysis was performed to further assess the effects of airway management techniques on EA in the unmatched cohort.

**Results:**

EA occurred in 543 patients (52.8%). The EA incidence in the propensity score-matched cohort was significantly higher in the tracheal tube group than in the LMA group [74% (123/166) versus 16% (26/166); relative risk: 4.73, 95% CI: 3.31–6.88, *P* < 0.001]. In the unmatched cohort, only when ketamine was used alone for anesthesia induction, the use of a tracheal tube was significantly associated with EA, compared with LMA use (odds ratio: 7.94, 95% CI: 3.38–18.63, *P* < 0.001).

**Conclusion:**

LMA use is associated with a lower incidence of EA than tracheal tube use in pediatric ophthalmic surgery, particularly when anesthesia is induced with ketamine.

## 1 Introduction

Emergence agitation is a temporary state of extreme arousal in pediatric patients recovering from general anesthesia, typically presenting as inconsolability, restlessness, and uncoordinated movements (1, 2). Unlike hypoactive emergence delirium, which is characterized by impaired communication, underactivity, and delayed response to interaction (3), emergence agitation is highly distressing to caregivers and poses risks of self-injury, surgical site disruption, and accidental removal of medical devices. Its occurrence complicates immediate postoperative management and increase the need for sedatives or opioids, thereby prolonging recovery and hospital stay (4).

The incidence of emergence agitation in children varies widely (10-80%), depending on surgical type, anesthetic technique, patient characteristics, and diagnostic criteria (5, 6). Among surgical procedures, ophthalmic surgeries have consistently been identified as high-risk for emergence agitation (7, 8). The heightened vulnerability of this surgical population has been attributed to factors such as postoperative visual disturbances, pain, and anxiety. Perioperative variables, including sex, age, anesthetic agents, airway management devices, and surgical and anesthetic durations, have been suggested to influence the likelihood of emergence agitation (9–12); however, findings remain inconsistent across studies. Despite its clinical importance, previous studies examining the association between emergence agitation and airway management techniques during pediatric ophthalmic surgery have been limited by small sample sizes (13, 14). Therefore, large-scale studies systematically investigating the association between them in this population are warranted.

We aimed to investigate the effects of airway management techniques on emergence agitation, controlling for confounding perioperative variables. Additionally, we explored the overall incidence of emergence agitation to provide evidence for perioperative strategies to mitigate and manage this condition in children undergoing ophthalmic surgery under general anesthesia.

## 2 Materials and methods

This single-center retrospective study was approved by the Institutional Review Board of Daegu Catholic University Medical Center (DCUMC 2025-08-018). Written informed consent was waived because of the study’s retrospective design.

### 2.1 Inclusion and exclusion criteria

We included patients aged 3–7 years with American Society of Anesthesiologists (ASA) class I or II who underwent strabismus or epiblepharon surgery under general anesthesia with sevoflurane, requiring airway management with either a tracheal tube or laryngeal mask airway (LMA) between January 2014 and December 2022. The exclusion criteria were insufficient medical records; strabismus or epiblepharon surgery combined with other surgery types; the use of anesthetics or analgesics other than ketamine, midazolam, sevoflurane, and fentanyl; and surgery performed on the day of admission.

### 2.2 Anesthetic management

Patients were admitted the day before the surgery and fasted for at least 8 h starting at midnight before the procedure. Thirty min before departing for the operating room, an intravenous catheter was placed in the venous lumen of one extremity, and 1,000 mL of Plasma-Lyte was infused via the catheter at a rate of 10 mL/kg/h for 2 h, after which the rate was reduced to 1 mL/kg/h (15). No pharmacological premedications or non-pharmacological interventions were administered prior to peripheral intravenous catheter placement. Upon arrival at the preoperative waiting room, intravenous atropine (0.01 mg/kg) was administered, and general anesthesia was induced with ketamine (1–2 mg/kg) or midazolam (0.05–0.1 mg/kg) in the presence of a guardian. When both agents were simultaneously used, the required dose was halved. After loss of consciousness, patients were immediately transferred to the operating room with continuous airway and vital signs monitoring. Upon arrival, preoxygenation was performed with 6 L/min of oxygen via a mask, and standard monitoring was initiated, including peripheral oxygen saturation (SpO_2_), electrocardiogram, non-invasive blood pressure, bispectral index, and neuromuscular blockade. All parameters were monitored throughout the perioperative period, except bispectral index and neuromuscular blockade, which were monitored until airway device removal. Subsequently, fentanyl (1–2 μg/kg) was administered to provide analgesia for airway device placement.

In the tracheal tube group, rocuronium (1 mg/kg) was administered to facilitate tracheal intubation, once four uniform amplitudes were obtained with train-of-four stimulation after the supramaximal current was detected with consecutive single twitches applied to the ulnar nerve at the forearm. With loss of spontaneous breathing, the airway was managed with mask-fit ventilation. When no response to train-of-four stimulation was observed, the trachea was intubated with a laryngoscope. In the LMA group, the decision to administer rocuronium under neuromuscular blockade monitoring, and dose selection, was at the discretion of the attending anesthesiologist. An LMA selected based on the patient’s weight was placed when the jaw was relaxed enough to be unresponsive to jaw thrust, and spontaneous breathing had ceased. Tracheal tube and LMA sizes were selected based on patient ages (16) and manufacturer’s weight-based recommendations (17), respectively. If the size of the airway device was inappropriate, an adjacent size was used at the discretion of the attending anesthesiologist.

The patients’ lungs were mechanically ventilated when clear breathing sounds were heard from both lungs through a stethoscope. The tidal volume and respiratory rate were adjusted to maintain the end-tidal carbon dioxide between 35 and 40 mmHg. Using the airway device, 2–3% sevoflurane was administered to maintain general anesthesia by maintaining bispectral index values between 40 and 60. When ketamine, which produces unreliable bispectral index values (18) was used to induce general anesthesia, the depth of general anesthesia was assessed by clinical evaluation. Intraoperative fentanyl was administered at the discretion of the attending anesthesiologist to provide analgesia and attenuate hemodynamic responses to surgical stimulation. All surgeries were performed by two experienced ophthalmologists, each specializing exclusively in strabismus and epiblepharon surgery. At the end of surgery, 0.075 mg/kg ondansetron was administered to prevent postoperative nausea and vomiting. If rocuronium was used, 0.3 mg/kg pyridostigmine and 0.006 mg/kg glycopyrrolate were administered to reverse neuromuscular blockade and prevent pyridostigmine-induced muscarinic effects, respectively. Once spontaneous breathing and consciousness were fully restored with SpO_2_ > 95%, tidal volume > 6 mL/kg, and bispectral index values > 90, the airway device was removed, and the patient was transferred to the post-anesthetic care unit (PACU).

Upon arrival in the PACU, oxygen was administered via a nasal cannula at 6 L/min. SpO_2_ was continuously monitored, and non-invasive blood pressure was measured only when the patient could tolerate cuff inflation. Airway patency, respiratory and hemodynamic stability, consciousness levels, and postoperative complications were assessed at 5-min intervals using a standardized protocol. Our institution maintains a strict and systematic post-anesthetic care protocol where the Pediatric Anesthesia Emergence Delirium (PAED) score is routinely evaluated in real time by attending anesthesiologists in the PACU. A PAED score threshold exceeding 12 is utilized to diagnose emergence agitation (19–21). Emergence agitation was characterized by the presence of hyperactive behavioral disturbances, such as restlessness, inconsolability, and disorientation, observed in the absence of hypoactive features, such as impaired communication, underactivity, and delayed responsiveness to interaction (3, 22). When emergence agitation occurred, 1–2 μg/kg fentanyl was consistently administered as a first-line treatment. Patients were discharged from the PACU when a modified Aldrete score ≥ 9 was achieved (23).

### 2.3 Data extraction and verification

Retrospective data collection was conducted using our institution’s integrated Clinical Data Warehouse (CDW) system, a specialized data-mining platform that systematically extracts deeply structured clinical data, baseline demographics, and longitudinal records directly from the Electronic Medical Record (EMR) and Order Communication System (OCS). Although real-time PAED scoring guided clinical decisions in accordance with our institutional protocol, individual itemized numerical scores were not universally archived as discrete, searchable fields across the entire multi-year EMR historical database. Nonetheless, a formal clinical diagnosis of emergence agitation by the attending anesthesiologists consistently remained recorded in the EMR. In instances where the CDW flagged missing variables or incomplete records, two independent investigators manually reviewed the primary electronic medical source documents to cross-reference and retrieve the data. Patients with permanently unresolvable or missing data fields were rigorously excluded from the final cohort analysis.

### 2.4 Study outcome variables

The collected outcome variables included: (1) patient characteristics (age, sex, height, weight, body mass index, and ASA physical status); (2) incidence of emergence agitation; (3) airway management techniques (tracheal tube versus LMA); (4) time from surgery completion to airway device removal; (5) type of anesthesia induction agent; (6) type of surgery (strabismus versus epiblepharon surgery); (7) doses and frequency of fentanyl and rocuronium use; (8) duration of anesthesia, surgery, and PACU stay; (9) presence or absence of upper respiratory tract infection symptoms; (10) time elapsed from the baseline study date (number of days from January 1, 2014); and (11) fasting time (minutes elapsed from the initiation of mandatory fasting at midnight to anesthesia induction).

### 2.5 Statistical analysis

Continuous variables were considered non-normally distributed if either the results of the Kolmogorov–Smirnov or Shapiro–Wilk test were significant; otherwise, they were considered normally distributed. Normally distributed continuous variables are reported as mean ± standard deviation and compared between two groups using an independent two-sample Student’s *t*-test. Non-normally distributed data are presented as medians (Q1, Q3) and compared between the two groups using the Mann–Whitney U test. Median differences were calculated using Hodges–Lehmann estimates. Categorical variables are reported as the number of patients (percentage) and compared between the two groups using the chi-square test. For the two-by-two chi-square test, the chi-square statistic was continuity-corrected.

The baseline characteristics of the tracheal tube and LMA groups were matched at a 1:1 ratio using propensity score matching with nearest neighbor matching method. The caliper was set at 0.015 and matching was performed without replacement. Propensity scores were generated by multivariable logistic regression analysis using baseline characteristics as independent variables and the binary group variable as the dependent variable. Good balance between the two groups was achieved with a standardized mean difference of < 0.1 (24). The matched pairs were compared using the paired *t*-test for normally distributed continuous variables and the Wilcoxon signed-rank test for non-normally distributed variables. For categorical variables, McNemar test was applied to dichotomous data, while McNemar-Bowker test was used for variables with more than two levels (25). Relative risk (RR) for matched pairs was calculated as the ratio of event probabilities between the tracheal tube and LMA groups with 95% CIs estimated using Nam–Blackwelder Score (26).

**FIGURE 1:**
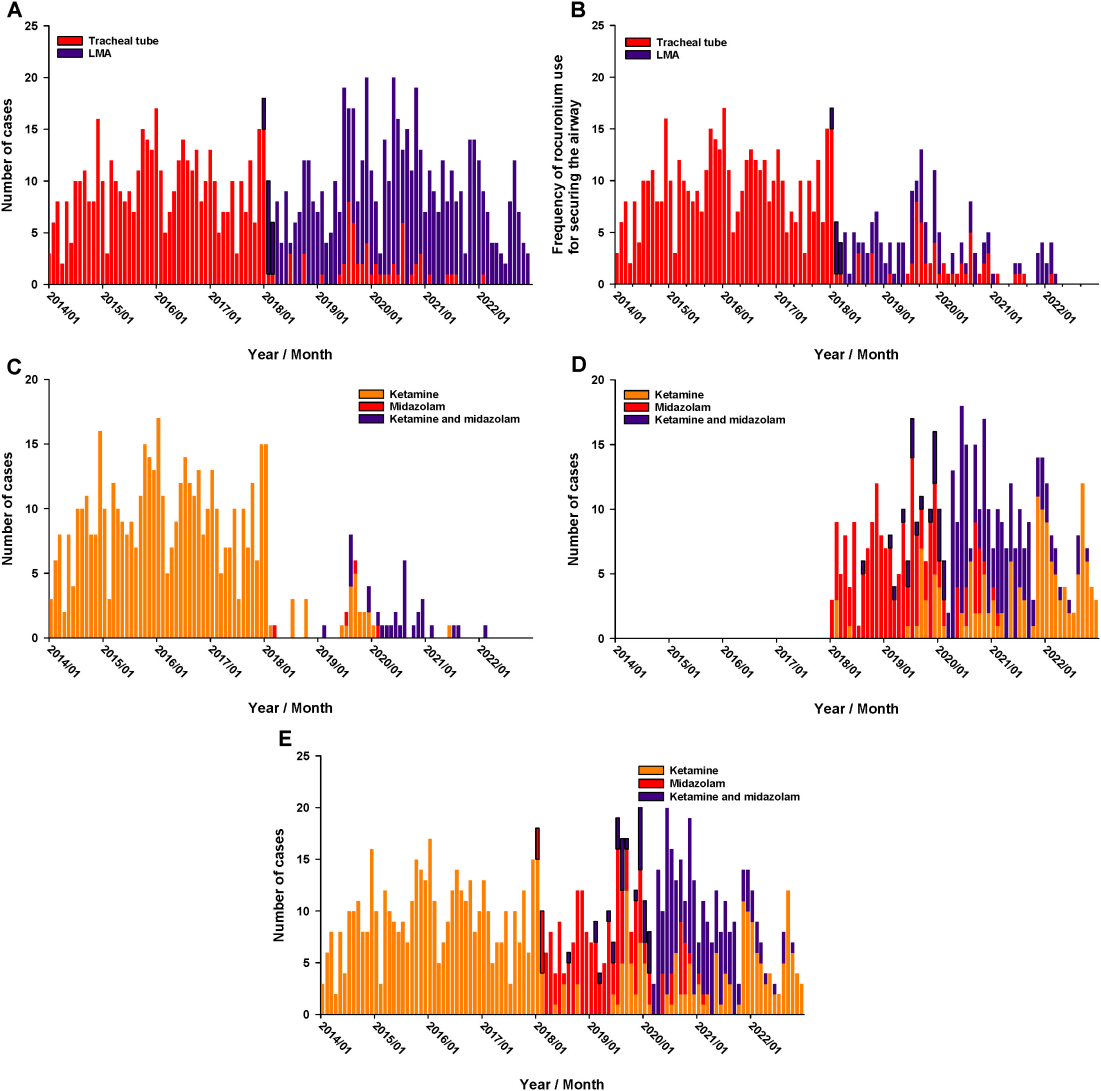
Chronological trends in airway management techniques, neuromuscular blocking agent, and anesthesia induction agents over the 9-year study period (from January 2014 to December 2022) in the unmatched population. **(A)** Longitudinal distribution of airway management techniques showing a significant institutional transition from conventional tracheal intubation to LMA placement around 2018. **(B)** Trends in the frequency of rocuronium use according to the airway management technique. **(C)** Trends in the selection of anesthesia induction agents within patients managed with a tracheal tube. **(D)** Trends in the selection of anesthesia induction agents within patients managed with an LMA. **(E)** Trends in the selection of anesthesia induction agents in the whole unmatched population. LMA, laryngeal mask airway.

Around 2018, our institution underwent a significant clinical transition in airway management practices, shifting from routine tracheal intubation to LMA utilization (27) (Figure 1). Because this structural shift created an unresolvable chronological imbalance between the two airway eras, attempting to balance the “time elapsed from the baseline study date” via propensity score matching would have drastically reduced the resulting sample size, thereby compromising statistical power. Therefore, this temporal variable was deliberately excluded from the matching process. Instead, to account for this unresolvable chronological imbalance, the “time elapsed from the baseline study date” was incorporated into the multivariable logistic regression model as an independent covariate, thereby mathematically adjusting for historic practice changes across the 9-year study period.

Factors contributing to emergence agitation were found using multivariable logistic regression analysis with the independent variables of (1) airway management techniques (tracheal tube versus LMA), (2) age, (3) sex (female versus male), (4) body mass index (5) ASA physical status (II versus I), (6) upper respiratory infection symptoms, (7) anesthesia induction agents (ketamine versus ketamine/midazolam and midazolam versus ketamine/midazolam), (8) time elapsed from the baseline study date, (9) fasting time, (10) surgery type (strabismus surgery versus epiblepharon surgery), (11) intraoperative fentanyl dose, (12) anesthesia duration, and (13) time from the end of the surgery to airway device removal. Because a significant association was observed between the airway management technique and the type of induction agent in the unmatched population (Table 1), the interaction effect between them was assessed in the multivariable logistic regression analysis. Variables causing multicollinearity (variance inflation factor > 5, and variance decomposition proportions > 0.8 with a condition index > 10) were excluded from the regression analysis (28).

**TABLE 1:** Baseline characteristics before and after propensity score matching.

	Before matching				After matching			
Variable	Tracheal tube (*n* = 524)	LMA (*n* = 505)	SMD	P	Tracheal tube (*n* = 166)	LMA (*n* = 166)	SMD	*P*
Age (years)	5.5 ± 1.1	5.3 ± 1.2	0.162	0.009	5.4 ± 1.1	5.4 ± 1.2	0.026	0.790
Sex			0.105	0.106			0.061	0.635
Male	265 (50.6)	229 (45.3)			74 (44.6)	69 (41.6)		
Female	259 (49.4)	276 (54.7)			92 (55.4)	97 (58.4)		
Height (cm)	114.8 ± 8.6	113.3 ± 9.9	0.160	0.011	113.6 ± 9.1	114.3 ± 9.0	0.070	0.470
Weight (kg)	22.5 ± 5.8	22.0 ± 5.9	0.099	0.112	21.9 ± 5.6	22.2 ± 5.8	0.052	0.605
BMI (kg/m^2^)	16.9 ± 2.7	16.9 ± 2.6	0.016	0.798	16.8 ± 2.5	16.8 ± 2.7	0.014	0.903
ASA physical status			0.119	0.087			0.042	>0.999
I	516 (98.5)	488 (96.6)			162 (97.6)	163 (98.2)		
II	8 (1.5)	17 (3.4)			4 (2.4)	3 (1.8)		
Presence of URI symptoms	30 (5.7)	22 (4.4)	0.063	0.390	6 (3.6)	8 (4.8)	0.060	0.774
Fasting time (min)	531.3 ± 76.7	542.7 ± 77.9	0.148	0.018	532.2 ± 72.0	533.3 ± 74.1	0.015	0.895
Induction agent			1.774	<0.001			0.037	0.368
Ketamine	490 (93.5)	148 (29.3)			137 (82.5)	136 (81.9)		
Midazolam	4 (0.8)	179 (35.4)			4 (2.4)	5 (3.0)		
Ketamine and midazolam	30 (5.7)	178 (35.2)			25 (15.1)	25 (15.1)		
Surgery type			0.137	0.033			0.012	>0.999
Epiblepharon surgery	280 (53.4)	304 (60.2)			102 (61.4)	103 (62.0)		
Strabismus surgery	244 (46.6)	201 (39.8)			64 (38.6)	63 (38.0)		
Doses of fentanyl used intraoperatively (μg/kg)	0.92 ± 0.30	0.85 ± 0.29	0.249	<0.001	0.81 ± 0.25	0.84 ± 0.34	0.085	0.427
Duration of anesthesia (min)	72.7 ± 20.3	65.9 ± 22.1	0.321	<0.001	65.8 ± 18.7	65.6 ± 19.7	0.010	0.911
Duration of surgery (min)	37.7 ± 18.4	32.6 ± 20.4	0.263	<0.001	32.2 ± 17.0	31.1 ± 16.6	0.068	0.440
Time elapsed from the baseline study date (days)	947.0 ± 598.5	2372.5 ± 482.9	2.621	<0.001	1153.5 ± 704.1	2684.0 ± 422.9	2.635	<0.001

Values are reported as mean ± SD or number of patients (percentage). LMA, laryngeal mask airway; SMD, standardized mean difference; BMI, body mass index; ASA, American Society of Anesthesiologists; URI, upper respiratory infection.

Statistical significance was defined as a two-tailed *P* < 0.05. All the statistical analyses were performed with the packages “tableone” and “MatchIt” for R (A Language and Environment for Statistical Computing, R Foundation for Statistical Computing)^1^ by R Core Team (2024), NCSS 2021 Statistical Software (2021) (NCSS, LLC., ncss.com/software/ncss), and IBM^®^ SPSS^®^ Statistics version 27 (IBM Corp.).

## 3 Results

### 3.1 Patient enrollment

**FIGURE 2:**
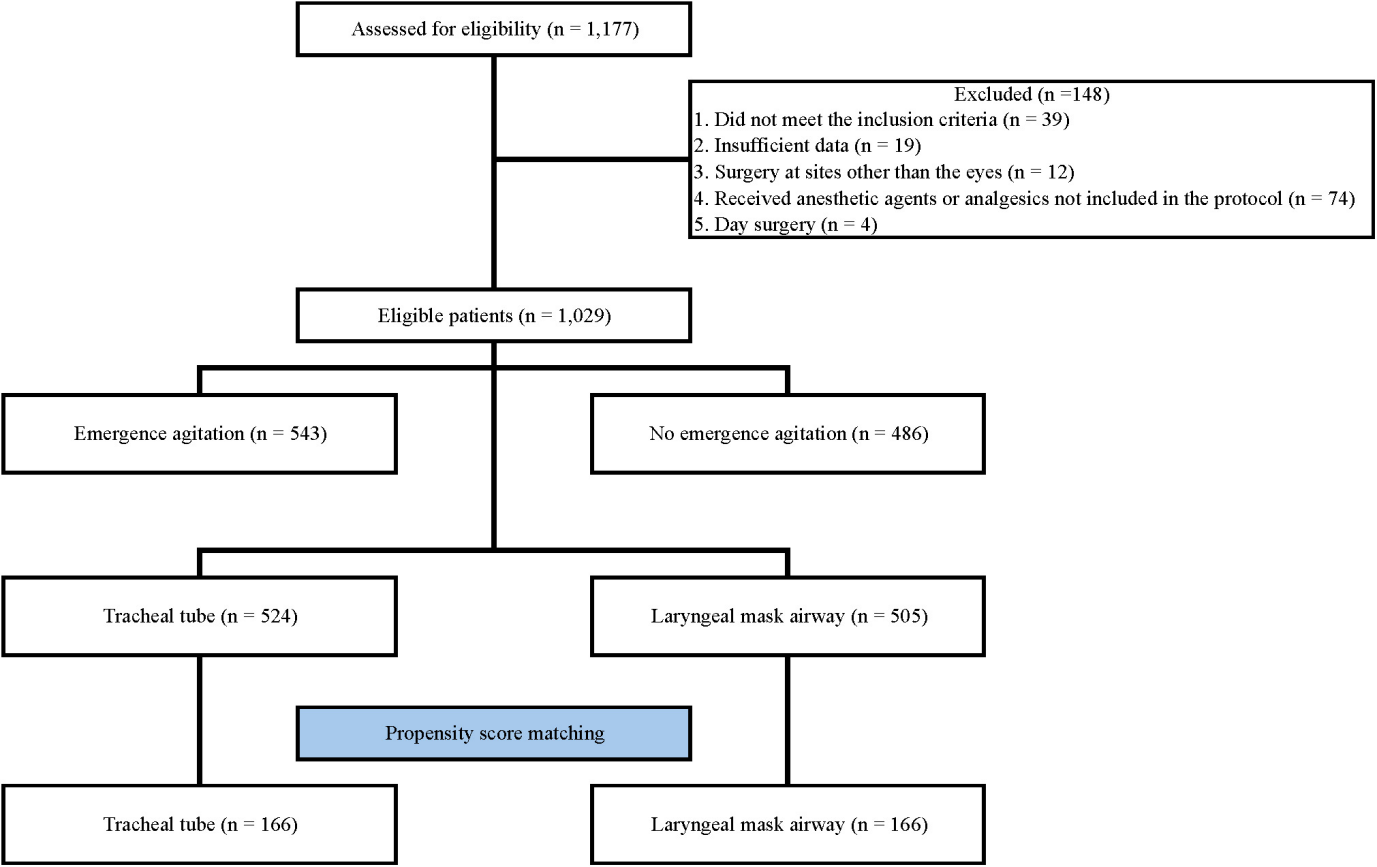
Flow diagram of patient selection, exclusion, airway management grouping, and propensity score matching. Of 1177 patients assessed, 1029 were included in the analysis. Emergence agitation occurred in 543 patients. Propensity score matching yielded 166 matched patients in each airway device group.

Of 1177 patients assessed for eligibility, 1029 were included in the analysis after excluding 148 patients for the following reasons: not meeting the inclusion criteria (*n* = 39), insufficient medical records (*n* = 19), combination of other surgery types involving sites other than the eyes (*n* = 12), use of anesthetic or analgesic outside the protocol (*n* = 74), and undergoing surgery on the same day of admission (*n* = 4) (Figure 2).

### 3.2 Propensity score matching

**FIGURE 3:**
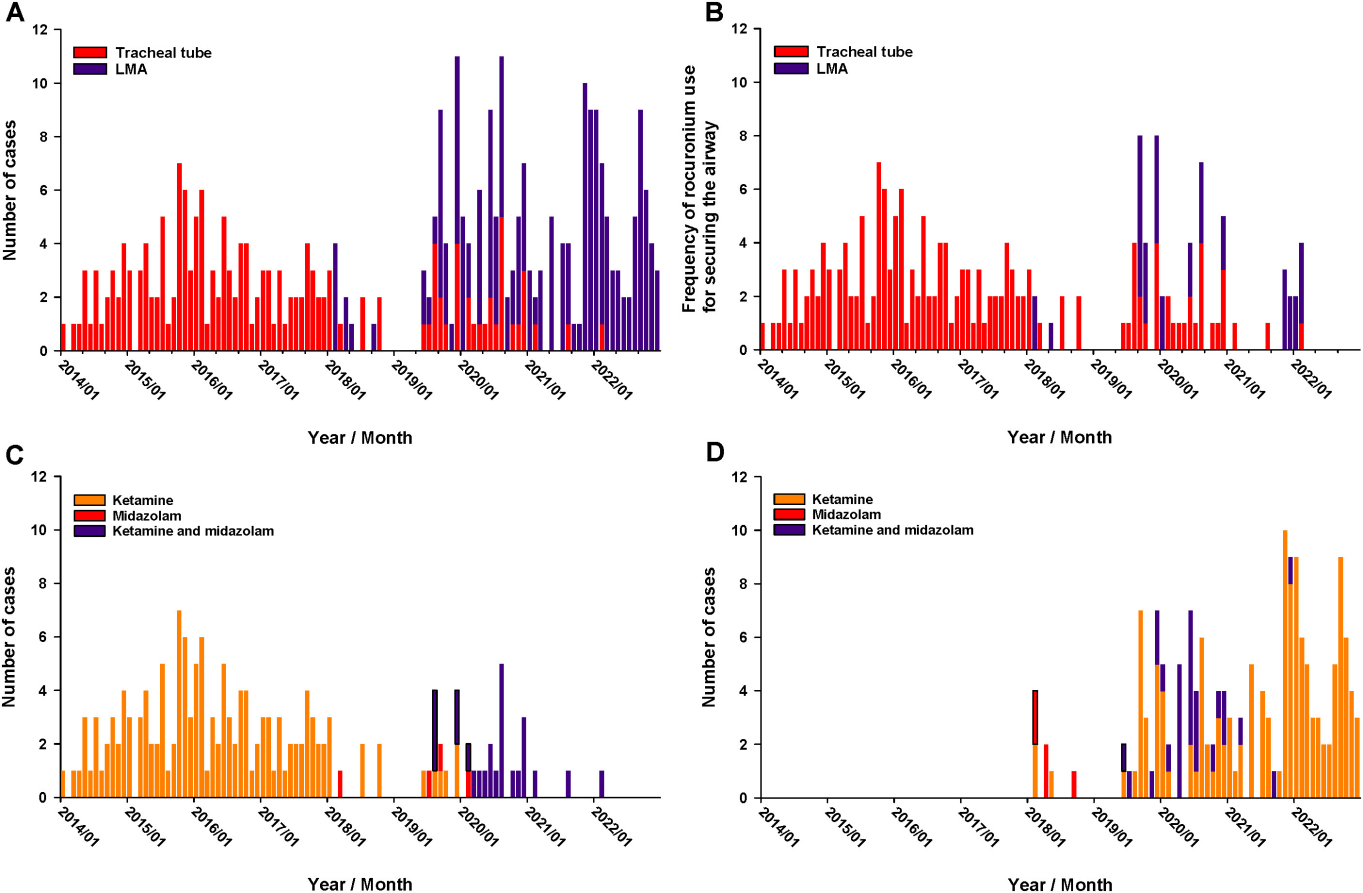
Chronological trends in airway management techniques, neuromuscular blocking agent, and anesthesia induction agents over the 9-year study period (from January 2014 to December 2022) in the matched population. **(A)** Longitudinal distribution of airway management techniques showing a significant institutional transition from conventional tracheal intubation to LMA placement around 2018. **(B)** Trends in the frequency of rocuronium use according to the airway management technique. **(C)** Trends in the selection of anesthesia induction agents within patients managed with a tracheal tube. **(D)** Trends in the selection of anesthesia induction agents within patients managed with an LMA. LMA, laryngeal mask airway.

Propensity score matching generated a well-balanced cohort with standardized mean differences < 0.1 between the tracheal tube and LMA groups (*n* = 166 per group) (Table 1). However, because the time elapsed from the baseline study date was intentionally excluded from the matching process, its standardized mean difference between the two groups remained > 0.1 after matching. The chronological distributions of the airway management techniques, rocuronium use, and selection of anesthesia induction agents resulting from this timeline imbalance are visualized in Figure 3. Before and after matching, compared with the LMA group, the tracheal tube group experienced emergence agitation more frequently [77.3% (405/524) versus 27.3% (138/505), RR (95% CI): 2.83 (2.44–3.29), *P* < 0.001 before matching; 74.1% (123/166) versus 15.7% (26/166), RR (95% CI): 4.73 (3.31–6.88), *P* < 0.001 after matching] and received rocuronium (before placing an airway device) (*P* < 0.001) and fentanyl (at the PACU) more frequently (*P* < 0.001) and at higher doses (*P* < 0.001 for rocuronium and fentanyl) (Table 2). In addition, the tracheal tube was extubated later than the LMA (*P* < 0.001 and *P* = 0.012, respectively). In the unmatched cohort, the length of PACU stay was longer (*P* < 0.001) in the tracheal tube group than in the LMA group; however, the between-group difference was not significant in the matched cohort.

**TABLE 2:** Perioperative data before and after propensity score matching.

	Before matching				After matching			
Variable	Tracheal tube (*n* = 524)	LMA (*n* = 505)	Median difference or relative risk (95% CI)	*P*	Tracheal tube (*n* = 166)	LMA (*n* = 166)	Median difference or relative risk (95% CI)	*P*
Number of patients with emergence agitation	405 (77.3)	138 (27.3)	2.83 (2.44–3.29)	<0.001	123 (74.1)	26 (15.7)	4.73 (3.31–6.88)	<0.001
Time from the end of the surgery to airway device removal (min)	10.0 (7.0, 13.8)	10.0 (6.0, 13.0)	1.0 (1.0–2.0)	<0.001	10.0 (7.0, 13.0)	9.0 (6.0, 13.0)	1.5 (0.5–2.0)	0.012
Frequency of fentanyl use at PACU				<0.001				<0.001
0	119 (22.7)	367 (72.7)	NA		43 (25.9)	140 (84.3)	NA	
1	386 (73.7)	118 (23.4)	NA		120 (72.3)	22 (13.3)	NA	
≥2	19 (3.6)	20 (4.0)	NA		3 (1.8)	4 (2.4)	NA	
Total doses of fentanyl used at PACU (μg/kg)	0.53 (0.31, 0.79)	0.00 (0.00, 0.35)	0.46 (0.42, 0.48)	<0.001	0.50 (0.00, 0.72)	0.00 (0.00, 0.00)	0.44 (0.33–0.50)	<0.001
Length of PACU stay (min)	47.0 (38.3, 57.0)	43.0 (34.0, 52.0)	4.0 (3.0–6.0)	<0.001	46.0 (37.0, 57.0)	46.0 (37.0, 53.3)	1.5 (–1.5 to 4.0)	0.339
Rocuronium use for securing the airway	520 (99.2)	119 (23.6)	4.21 (3.61–4.95)	<0.001	164 (98.8)	43 (25.9)	3.81 (2.99–4.98)	<0.001
Doses of rocuronium used to secure the airway (mg/kg)	0.71 (0.57, 0.88)	0.00 (0.00, 0.00)	0.64 (0.63–0.66)	<0.001	0.63 (0.52, 0.77)	0.00 (0.00, 0.17)	0.56 (0.51, 0.60)	<0.001

Values are reported as median (Q1, Q3) or number of patients (percentage). LMA, laryngeal mask airway; PACU, post-anesthetic care unit; NA, not applicable.

### 3.3 Univariate analysis for emergence agitation

Among the 1029 patients, 543 (52.8%) experienced emergence agitation. In the univariate analysis (Table 3), significant odds of developing emergence agitation were demonstrated in patients who underwent tracheal intubation requiring rocuronium (versus LMA), strabismus surgery (versus epiblepharon surgery), or prolonged anesthesia and surgery at an earlier date; were male; were administered a single anesthesia induction agent (versus a combination of ketamine and midazolam); fasted for a short duration. Patients who developed emergence agitation remained in the PACU longer and required more fentanyl than those who did not.

**TABLE 3:** Univariate analysis of baseline characteristics and perioperative data in patients with and without emergence agitation in the PACU.

Variable	Emergence agitation (*n* = 543)	No emergence agitation (*n* = 486)	Mean/median difference or odds ratio (95% CI)	*P*
Airway management				
LMA	138 (25.4)	367 (75.5)	Reference	NA
Tracheal tube	405 (74.6)	119 (24.5)	9.05 (6.82–12.01)	<0.001
Age (years)	5.4 ± 1.1	5.4 ± 1.2	0.03 (–0.12 to 0.17)	0.723
Sex				
Male	281 (51.7)	213 (43.8)	Reference	NA
Female	262 (48.3)	273 (56.2)	0.72 (0.57–0.93)	0.013
Height (cm)	114.0 ± 8.8	114.1 ± 9.8	–0.1 (–1.2 to 1.0)	0.863
Weight (kg)	22.1 ± 5.6	22.5 ± 6.1	–0.42 (–1.14 to 0.30)	0.255
BMI (kg/m^2^)	16.8 ± 2.7	17.0 ± 2.6	–0.16 (–0.54 to 1.06)	0.187
ASA physical status				
I	532 (98.0)	472 (97.1)	Reference	NA
II	11 (2.0)	14 (2.9)	0.70 (0.31–1.55)	0.492
Presence of URI symptoms	32 (5.9)	20 (4.1)	1.46 (0.82–2.59)	0.247
Time elapsed from the baseline study date (days)	1033.0 (624.0, 1709.0)	2351.0 (1791.0, 2695.5)	–1128.0 (–1222.0 to –1030.0)	<0.001
Fasting time (min)	492.0 (485.0, 577.0)	495.0 (488.0, 578.3)	–5.0 (–9.0 to –2.0)	<0.001
Induction agent				
Ketamine and midazolam	46 (8.5)	162 (33.3)	Reference	NA
Ketamine	419 (77.2)	219 (45.1)	6.74 (4.67–9.72)	<0.001
Midazolam	78 (14.4)	105 (21.6)	2.62 (1.69–4.06)	<0.001
Surgery type				
Epiblepharon surgery	292 (53.8)	292 (60.1)	Reference	NA
Strabismus surgery	251 (46.1)	194 (39.9)	1.29 (1.01–1.66)	0.048
Doses of fentanyl used intraoperatively (μg/kg)	0.89 ± 0.27	0.89 ± 0.33	–0.003 (–0.40 to 0.03)	0.881
Duration of anesthesia (min)	72.4 ± 22.3	66.1 ± 19.9	6.29 (3.69–8.89)	<0.001
Duration of surgery (min)	38.2 ± 20.9	31.9 ± 17.4	6.31 (3.96–8.65)	<0.001
Rocuronium use for securing the airway	447 (82.3)	192 (39.5)	7.13 (5.36–9.49)	<0.001
Doses of rocuronium used to secure the airway (mg/kg)	0.63 (0.37, 0.83)	0.00 (0.00, 0.38)	0.47 (0.40–0.50)	<0.001
Time from the end of the surgery to airway device removal (min)	9.0 (7.0, 13.0)	10.0 (7.0, 14.0)	–1.0 (–1.0 to 0.0)	0.078
Frequency of fentanyl use at PACU				<0.001
0	0 (0.0)	486 (100.0)	NA	NA
1	504 (92.8)	0 (0.0)	NA	NA
≥2	39 (7.2)	0 (0.0)	NA	NA
Total doses of fentanyl used at PACU (μg/kg)	0.57 (0.48, 0.87)	0.00 (0.00, 0.00)	0.57 (0.56–0.59)	<0.001
Length of PACU stay (min)	48.0 (39.0, 58.0)	42.0 (34.0, 52.0)	6.0 (4.0–7.0)	<0.001

Values are reported as mean ± SD, median (Q1, Q3), or number of patients (percentage). LMA, laryngeal mask airway; NA, not applicable; BMI, body mass index; ASA, American Society of Anesthesiologists; URI, upper respiratory infection; PACU, post-anesthetic care unit; UA, unavailable.

### 3.4 Multivariable logistic regression for emergence agitation

A significant interaction effect was observed between the airway management technique and the anesthesia induction agents in the multivariable logistic regression model (Table 4). When ketamine was used alone for anesthesia induction, the use of a tracheal tube was significantly associated with development of emergence agitation compared with an LMA [odds ratio (OR): 7.94, 95% CI: 3.38–18.63, *P* < 0.001]. Conversely, when a tracheal tube was used for airway management, the use of ketamine alone significantly increased the odds of developing emergence agitation compared with the combined use of ketamine and midazolam (OR: 7.70, 95% CI: 2.25–26.34, *P* < 0.001).

**TABLE 4:** Multivariable logistic regression analysis of baseline characteristics and perioperative data in patients with and without emergence agitation in the PACU.

Variable	Odds ratio (95% CI)	*P*
Interaction effect between airway management technique and anesthesia induction agent*	**NA**	**NA**
Ketamine/midazolam	NA	NA
Tracheal tube versus LMA	0.76 (0.29–2.04)	0.588
Ketamine	NA	NA
Tracheal tube versus LMA	7.94 (3.38–18.63)	<0.001
Midazolam	NA	NA
Tracheal tube versus LMA	1.79 (0.24–13.39)	0.569
LMA	NA	NA
Ketamine versus ketamine/midazolam	0.74 (0.38–1.45)	0.507
Midazolam versus ketamine/midazolam	1.82 (0.99–3.32)	0.054
Tracheal tube	NA	NA
Ketamine versus ketamine/midazolam	7.70 (2.25–26.34)	<0.001
Midazolam versus ketamine/midazolam	4.28 (0.36–50.95)	0.328
Age (years)	0.95 (0.82–1.10)	0.524
Sex	NA	NA
Female versus male	0.79 (0.58–1.07)	0.133
BMI (kg/m^2^)	0.98 (0.92–1.04)	0.428
ASA physical status	NA	NA
II versus I	1.24 (0.43–3.51)	0.693
Presence of URI symptoms	1.25 (0.65–2.47)	0.508
Time elapsed from the baseline study date (days)	0.9993 (0.9989–0.9996)	<0.001
Fasting time (min)	1.00 (1.00–1.00)	0.583
Surgery type	NA	NA
Strabismus surgery versus epiblepharon surgery	0.88 (0.57–1.35)	0.549
Doses of fentanyl used intraoperatively (μg/kg)	0.26 (0.14–0.45)	<0.001
Duration of anesthesia (min)^†^	1.01 (1.00–1.02)	0.049
Time from the end of the surgery to airway device removal (min)	0.96 (0.94–0.99)	0.006

*The variables for rocuronium use were not included in the analysis because their correlation with the airway management method could lead to multicollinearity.

† Duration of surgery was not included in the analysis because its correlation with duration of anesthesia could lead to multicollinearity. The variables for postoperative fentanyl use and length of PACU stay were not included in the analysis because they were made by the consequent effects of the dependent variable (emergence agitation versus no emergence agitation). In other words, they do not affect the dependent variable. LMA, laryngeal mask airway; NA, not applicable; BMI, body mass index; ASA, American Society of Anesthesiologists; URI, upper respiratory infection; PACU, post-anesthetic care unit.

One-unit increase in intraoperative fentanyl dose (μg/kg), time elapsed from the baseline study date (days), time from the end of surgery to airway device removal (min), and anesthesia duration (min) significantly contributed to 74% decrease (*P* < 0.001), 0.07% decrease (*P* < 0.001), 4% decrease (*P* = 0.006), and 1% increase (*P* = 0.049) in the odds to develop emergence agitation, respectively, assuming that all the other independent variables were held constant.

## 4 Discussion

In this retrospective study, emergence agitation occurred in over half of the study population (52.8%) who underwent strabismus or epiblepharon surgery under general anesthesia. Patients undergoing tracheal intubation experienced emergence agitation and required postoperative fentanyl more frequently than those undergoing LMA insertion according to the result of propensity score matching analysis. In addition, the LMA was removed from the airway earlier than the tracheal tube. Multivariable logistic regression demonstrated that tracheal intubation was more strongly associated with emergence agitation than the use of an LMA, only when ketamine was administered for anesthesia induction. Shorter time to airway device removal, lower doses of intraoperative fentanyl, longer anesthetic duration, and earlier surgery date were independently associated with emergence agitation. Affected patients required longer postoperative care, as reflected by a prolonged PACU stay. These findings suggest that airway management and anesthetic techniques play important roles in the development of emergence agitation in children undergoing ophthalmic surgery.

Ophthalmic surgery is a known major predictor of emergence agitation (7). However, to our knowledge, only one randomized controlled trial (RCT) (13) and one retrospective study (14) have compared emergence agitation between tracheal tubes and LMAs in pediatric patients. The RCT demonstrated a lower incidence of emergence agitation with LMA use compared with a tracheal tube between 5 and 15 min after arrival at the PACU with the most significant difference observed at 10 min (11.1% versus 37.8%, respectively) (13). These incidence rates are notably lower than those observed in our unmatched cohort (27.3% versus 77.3%). In contrast, the retrospective study found no significant difference in PAED scores between the devices, although the incidence of emergence agitation was not available (14). Notably, the reliability of these previous findings is limited. The sample size calculation in the RCT is not reproducible based on the provided parameters (13), and the retrospective study was likely underpowered due to a small sample size (*n* = 23 and *n* = 21 per group) (14). In contrast, a major strength of the current study is the significantly larger sample size (*n* = 166 per group), affording much greater statistical power to detect true differences between the airway devices.

Laryngoscopy exerts great pressure on the tongue and epiglottic fold. Subsequent tracheal intubation stimulates the laryngeal and tracheal mucosa, and the pressure from cuff inflation (29) stimulates the tracheal mucosa further, thereby overactivating the sympathetic nervous system (30). While in place, the tracheal tube continues to irritate the airway. During emergence, airway reflexes recovered by the reversal of neuromuscular blockade move the tracheal tube, further irritating the sensitized, edematous airways. The resulting postintubation sore throat (31) is a clinical entity that may contribute to the development of emergence agitation.

In contrast, LMA insertion and removal typically causes less mucosal trauma because there is no chance of irritating the larynx and trachea, and LMA inflation does not produce as much pressure on the pharynx as laryngoscopy. Furthermore, neuromuscular blocking agents are required less frequently with an LMA than with a tracheal tube; a previous retrospective study reported that only 27.5% (29/142) of patients in the LMA group required neuromuscular blockade, whereas all 68 patients in the tracheal group required it (32). Consistent with these findings, we observed that LMA use required neuromuscular blocking agents less frequently and at lower doses than tracheal intubation. Minimizing the use of neuromuscular blocking agents facilitates earlier airway device removal, thereby reducing airway stimulation during the emergence phase.

Because postoperative pain scores, such as Face, Legs, Activity, Cry, and Consolability (FLACC) or Wong-Baker Faces scales, were not assessed in our study, it remains unclear whether or to what extent differences in postoperative pain levels or individual physiological responses between the two airway management methods may have influenced our results. Within our propensity score-matched cohort, the incidence of emergence agitation was 15.7% in the LMA group versus 74.1% in the tracheal tube group. Furthermore, multivariable logistic regression identified the airway management method as the independent predictor strongly associated with emergence agitation development, yielding the highest OR (7.94) among all included variables. Regrettably, previous literature also demonstrates inconsistent results regarding the relationship between pain and airway management techniques. A previous RCT involving children undergoing strabismus surgery reported a significant correlation between PAED and FLACC scores and significant differences in both scores and emergence agitation incidence between the two groups (11.5% versus 37.8% at 10 min post-arrival in the PACU) (13). Similarly, an RCT in pediatric dental rehabilitation showed a significantly lower incidence of emergence agitation (5.7% versus 100%) with reduced laryngeal pain measured via the Wong-Baker Faces scale in the LMA group (33). In contrast, while an RCT for adenoidectomy also demonstrated a lower incidence of emergence agitation in the LMA group compared with the tracheal tube group (15.4% versus 41.0%), it found no significant difference in the FLACC score between the two groups (34).

The RR, defined as the ratio of incidence rates between two groups (35), varied widely across the literature. In our study, propensity score matching adjusted for baseline covariates, shifting the crude RR of emergence agitation between the tracheal tube and LMA groups from 2.83 to an adjusted RR of 4.73. In comparison, the RRs from previous RCTs were 3.41 [ = (14/37)/(4/36)] for strabismus surgery (13), 17.5 [ = (35/35)/(2/35)] for dental rehabilitation (33), 2.67 [ = (16/39)/(6/39)] for adenoidectomy (34), and 1.92 [ = (23/56)/(12/56)] for subumbilical surgery (11). This heterogeneity likely stems from variations in surgical techniques, differing anesthetic management protocols, and diverse pediatric populations. In addition, differences in grouping methodology (randomization in the previous trials versus retrospective propensity score matching in our study) alongside the residual confounding inherent to our retrospective study design might have further contributed to the variance in these estimated effect sizes. Unlike our study, two of these studies (13, 34) used propofol, and one of them (11) used propofol and/or fentanyl to manage emergence agitation, while the remaining study did not have data on rescue medications (33).

We found a significant interaction between the choice of induction agent and the type of airway device in the development of emergence agitation. Specifically, while ketamine is widely recognized for its analgesic properties (36–38), which may contribute to the prevention of emergence agitation (39), it appears to have a paradoxical unfavorable effect when a tracheal tube is used. However, these negative effects of ketamine were minimal in patients where an LMA was used for airway management. It is speculated that the dissociative state achieved by ketamine (38, 40) is aggravated by the airway irritation and sympathetic overactivation resulting from tracheal intubation, thereby leading to emergence agitation. In contrast, the more stable physiological environment provided by an LMA does not worsen the side effects of ketamine.

In contrast to the variable performance of ketamine, the addition of midazolam to the anesthesia induction regimen was consistently associated with a low risk of emergence agitation, irrespective of the airway device employed. Its beneficial effects have also been demonstrated in a recent network meta-analysis (41). Although midazolam can cause paradoxical reactions (restlessness, disorientation, and inconsolable crying) that mimic emergence agitation (42), we speculate that its potent anxiolytic and sedative properties reduced preoperative anxiety, which is one of the risk factors for emergence agitation (36, 37). In addition, the use of midazolam reduces analgesic requirements (41). Based on our findings, the combined use of midazolam and ketamine seemed to minimize their respective side effects and produced a synergy of their beneficial effects.

This study has some limitations. First, as emergence agitation and emergence delirium share common characteristics (36, 37), vigilance should be exercised in distinguishing between them. However, the retrospective nature of this study does not guarantee that the attending anesthesiologist perfectly adhered to our criteria for emergence agitation to detect changes in attention and awareness, which are important features of emergence delirium, or to distinguish hypoactive delirium from a quiet state with intact cognition. Second, as a single-center retrospective study, our findings may not be generalizable to other institutions with different anesthetic protocols or postoperative care standards. In addition, although propensity score matching was performed to evaluate the effects of airway devices on emergence agitation, the retrospective design of this study could not assess the cause-and-effect relationship. Third, potential confounding factors, particularly psychological variables, such as preoperative anxiety levels in children or caregivers, were not included in the analysis, which may have influenced our results. In addition, although postoperative pain is closely related to emergence agitation, clinically distinguishing these two entities remains an ongoing scientific challenge in pediatric anesthesia research (43, 44). Indeed, one prospective observational study reported that up to 65% of pediatric patients exhibited signs of both emergence agitation and postoperative pain simultaneously, making it difficult to separate their individual pathways (44). Nonetheless, the postoperative pain was not formally assessed or analyzed in this study. Therefore, its specific contribution to the outcome variable could not be accurately quantified. Furthermore, we cannot completely exclude the presence of other unknown or unmeasured confounders that might have affected our results. Fourth, although we demonstrated the association of LMA use with a low incidence of emergence agitation, its limitations, which significantly affect patient safety, should always be considered (including placement failure requiring tracheal intubation (45, 46) and the risk of gastric content aspiration (45)). Fifth, our findings that the use of ketamine in patients undergoing tracheal intubation were associated with a higher risk of emergence agitation warrant caution due to the retrospective nature of this study. Because the choice and dosing of induction agents were left entirely to the discretion of the attending anesthesiologists rather than being randomly assigned, we cannot completely rule out selection bias or confounding by indication. Furthermore, while we speculated that airway irritation from tracheal intubation amplifies the dissociative effects of ketamine, our retrospective data lacks metrics for the depth of anesthesia or plasma drug concentration profiles to definitively confirm this precise mechanistic interplay. Prospective RCTs standardizing both the airway management technique and the induction regimen are necessary to conclusively delineate how specific induction agents independently influence emergence agitation in this population. Finally, there was a disagreement in the results for the time to remove the airway device between the multivariable logistic regression and the comparison between the two airway devices in the propensity score-matched population. Although a decrease in the time was associated with the development of emergence agitation according to the logistic regression model, the tracheal tube group, with a higher incidence of emergence agitation (versus the LMA group), spent more time extubating. The disagreement in the results cannot be fully explained, as it depends only on our data.

In summary, LMA use is associated with a low incidence of emergence agitation in preschool children undergoing ophthalmic surgery under sevoflurane anesthesia. Given the clinical burden associated with emergence agitation, including increased PACU interventions, delayed discharge, and heightened caregiver distress, strategies that reduce its incidence may contribute to a safer and smoother postoperative recovery. Further prospective multicenter studies are warranted to validate these findings and to develop standardized protocols for high-risk pediatric populations.

## Data Availability

The raw data supporting the conclusions of this article will be made available by the authors, without undue reservation.

## Footnotes

1
https://www.R-project.org
